# Prophage-mediated genome differentiation of the *Salmonella* Derby ST71 population

**DOI:** 10.1099/mgen.0.000817

**Published:** 2022-04-22

**Authors:** Jinyan Yu, Xiaomeng Xu, Yu Wang, Xianyue Zhai, Zhiming Pan, Xinan Jiao, Yunzeng Zhang

**Affiliations:** ^1^​ Jiangsu Co‐Innovation Center for Prevention and Control of Important Animal Infectious Diseases and Zoonoses, Yangzhou University, Yangzhou 225009, PR China; ^2^​ Jiangsu Key Laboratory of Zoonosis, Yangzhou University, Yangzhou 225009, PR China; ^3^​ Joint International Research Laboratory of Agriculture and Agri‐product Safety of the Ministry of Education, Yangzhou University, Yangzhou 225009, PR China; ^4^​ Key Laboratory of Prevention and Control of Biological Hazard Factors (Animal Origin) for Agrifood Safety and Quality, Ministry of Agriculture of China, Yangzhou University, Yangzhou 225009, PR China

**Keywords:** *Salmonella *Derby, prophage, swine, poultry, pan-genome

## Abstract

Although *Salmonella* Derby ST71 strains have been recognized as poultry-specific by previous studies, multiple swine-associated *S*. Derby ST71 strains were identified in this long-term, multi-site epidemic study. Here, 15 representative swine-associated *S*. Derby ST71 strains were sequenced and compared with 65 (one swine-associated and 64 poultry-associated) *S*. Derby ST71 strains available in the NCBI database at a pangenomic level through comparative genomics analysis to identify genomic features related to the differentiation of swine-associated strains and previously reported poultry-associated strains. The distribution patterns of known *Salmonella* pathogenicity islands (SPIs) and virulence factor (VF) encoding genes were not capable of differentiating between the two strain groups. The results demonstrated that the *S*. Derby ST71 population harbours an open pan-genome, and swine-associated ST71 strains contain many more genes than the poultry-associated strains, mainly attributed to the prophage sequence contents in the genomes. The numbers of prophage sequences identified in the swine-associated strains were higher than those in the poultry-associated strains. Prophages specifically harboured by the swine-associated strains were found to contain genes that facilitate niche adaptation for the bacterial hosts. Gene deletion experiments revealed that the *dam* gene specifically present in the prophage of the swine-associated strains is important for *S*. Derby to adhere onto the host cells. This study provides novel insights into the roles of prophages during the genome differentiation of *Salmonella *.

## Data Summary

**Impact Statement**

Previous studies have suggested that prophages are crucially involved in diversifying the genome sequences and host ranges of *Salmonella* as evidenced based on analysis of the relatively ancient differentiated strains (i.e. different species, subspecies, or serotypes). However, information about the roles of prophages in genome differentiation of *Salmonella* strains with very-closely conserved genomic backgrounds is still lacking (e.g. strains affiliated with the same ST schema). Here, we demonstrated that prophages function as important drivers to divide the *S*. Derby ST71 population into two distinct groups with different host ranges. The swine-associated ST71 strains were found to harbour significantly more prophages contributing to larger genome sizes, higher number of genes and lower GC content in this group compared with those in the poultry-associated ST71 strains. We also identified several cargo genes contained in the prophages that can facilitate niche adaptation of the bacterial hosts. Gene deletion experiments revealed that the *dam* gene contained in the prophages specific to swine-associated strains is essential for *S*. Derby to adhere onto the host cells. This study provides novel insights about the roles of prophages in the genome differentiation of closely-related *Salmonella* populations.

The genome sequences of the 15 *S*. Derby strains have been deposited in CNGB database under project CNP0002351, and other strains were from NCBI Refseq database under BioProject No. PRJNA391404 and PRJNA464303, and assembly GCF_002063355.1.

## Introduction

*Salmonella enterica* subspecies *enterica* serovar Derby (*S*. Derby) is reported to be among the most prevalent *Salmonella* serotypes isolated from pigs and poultry. It is recognized as an important zoonotic pathogen that mainly affects at-risk groups including infants, children, and elderly people [1–4]. Of the 26 multi-locus sequence type (MLST) profiles (STs) identified in this serovar, ST40, ST71, ST682, and ST39 are the most frequently recorded STs in the Enterobase *Salmonella* MLST database (https://enterobase.warwick.ac.uk/species/index/senterica) (accessed on 14 September 2021) and pubMLST (accessed on 22 August 2021) [5]. Although ST40, ST39, and ST682 have been reported to be predominantly associated with swine, a strong link has been suggested between ST71 and poultry based on previous reports and the Enterobase database; these poultry-specific ST71 strains were mainly isolated in Europe, including France and Germany [6–8]. In China, ST40 and ST71 were the two main MLST profiles associated with *S*. Derby [9]. ST40-affiliated strains identified in China were mainly isolated from swine-associated samples, which was consistent with previous findings in Europe and other geographic locations. Although our laboratory isolated multiple ST71 strains from swine-associated samples, no poultry-originating ST71 strain was isolated in this long-term multi-site epidemic study (2013–2015), even after screening numerous poultry-associated samples [2]. *S*. Derby ST71 strains have also been isolated from swine-associated samples in China by other researchers; for instance, *S*. Derby BCW_2839 (refSeq GCF_002063355.1) was a strain isolated from a pork sample sold in a rural market, Jiyuan, China in 2006 (Dr. Kjeldgaard, personal communications).

*S. enterica* is recognized as a complex species that harbours diversified genomes with distinct host range variations [10, 11]. Horizontal gene transfer (HGT), mediated by the transfer of plasmids, transposons, and phages [12], is among the main driving forces in the diversification and niche adaptation process of *Salmonella* [13]. Acquisition of SPI-1 (* Salmonella* pathogenicity island 1) through HGT is considered the main evolutionary event that allowed *Salmonella* to be differentiated from closely-related *Escherichia coli *, and the subsequent acquisition of SPI-2 further caused divergence of *S. enterica* from *S. bongori *; then, *S. enterica* continued to differentiate into seven subspecies and more than 2600 serotypes with varying host adaptation or restriction patterns based on their acquisition of SPIs in addition to other evolutionary events [14]. Notably, incorporation of the prophage genes into the accessory genomic content is crucial in the *Salmonella* diversification progress, and these imported prophage sequences can effectively distinguish between *Salmonella* populations at a high resolution, even at the strain level [15]. Phages, or bacteriophages, are the most abundant organisms in the biosphere; therefore, they can infect bacteria and integrate their DNA into the bacterial chromosome to form a prophage, with the integrated prophage sequences, accounting for 3–10 % of the whole bacterial genomic content [16]. Furthermore, prophages with highly similar DNA sequences can function as anchoring points for homologous recombination reactions, leading to major genomic rearrangement and genomic content exchange [16]. In fact, acquired prophages can modify the structure and functions of a bacterial community, thereby creating a selective advantage for bacteria to adapt to diverse environments through prophage-mediated import of functional genes; therefore, they are crucially involved in bacterial colonization, pathogenicity, and evolution [16, 17]. Numerous studies have demonstrated that prophage-mediated gene acquisition events endow *Salmonella* with abilities to colonize the host and compete with its competitors [18–20]. Although several diverse prophage sequences have been identified in multiple *Salmonella* serotypes [21], the extent and manner in which prophages affect genome diversification and the host adaptation properties of *Salmonella* populations with highly-conserved genomic backgrounds (e.g. strains affiliated with the same ST scheme) remain largely unknown.

To date, multiple poultry-specific *S*. Derby ST71 strains have been sequenced; however, genome sequencing data for swine-associated ST71 strains are still lacking, which limits the identification of specific genomic signature patterns, such as prophage sequences, between two strain groups that are associated with observable host range differences. In this study, we selected 15 representative *S*. Derby ST71 strains based on CRISPR typing profiles [2, 22] from our large collection of *S*. Derby strains, containing more than 400 *S*. Derby strains isolated from multiple sites over several years; subsequently, whole genome sequencing was performed. All 15 *S*. Derby ST71 strains were isolated from swine-associated samples between 2013 and 2015 (14 from pork samples and one from a slaughterhouse). One swine-associated *S*. Derby ST71 strain available in NCBI refseq database, BCW_2839 (refSeq GCF_002063355.1) was also included in this analysis. The genomic content differences between the 16 swine-associated strains and the 64 poultry-associated strains were compared using a pangenome-based comparative genomics analysis to identify the genomic contents that are associated with host differences between the two groups.

## Methods

### Whole genome sequencing of *Salmonella* Derby ST71 strains

Based on their CRISPR typing profiles [2, 22], 15 representative swine-associated *Salmonella* Derby ST71 strains were selected from our collection of *Salmonella* cultures, which were isolated from the Jiangsu province, China between 2013 and 2015. These strains were subjected to whole genome sequencing (Table S1, available in the online version of this article). Genomic DNA was extracted using the PureLink Genomic DNA kit (Invitrogen, USA). The quality of the DNA was examined by agarose gel electrophoresis (0.8 %), and then quantified using Agilent 2100 BioAnalyzer (Agilent Technologies, Inc., Santa Clara, CA, USA). Whole genome sequencing was performed using the Illumina HiSeq technology, which generated 2×150 bp paired-end reads. The reads were *de novo* assembled using SPAdes ver.3.11 with –careful and –cov cut-off auto options [23]. Only scaffolds longer than 200 bp were included.

### Annotation

All publicly available *S*. Derby genomes in the NCBI Refseq database were downloaded (accessed on 2 January 2020). Sequence types (STs) of the genomes were determined using the MLST software (ver. 2.19.0) based on the PubMLST database [24]. In total, 65 genomes associated with *S*. Derby ST71 (one swine-associated strain and 64 poultry-associated strains) (Table S1) were extracted for further analyses.

The *de novo* assembled and downloaded genomes were simultaneously annotated using PROKKA ver. 1.13 [25]. The Clusters of Orthologous Groups of proteins (COG) annotations were assigned to genes using eggNOG-mapper-2.0.1–14 [26] based on the eggNOG orthology data [27]. Diamond was used to perform sequence searches with the blastp option [28]. The prophage sequences were predicted using ProphET based on the implemented database downloaded on 16 April 2020 [29]. The prophage sequences were grouped into clusters using CD-hit-est, and sequences with 90 % sequence similarity were grouped into one cluster (parameter -c 0.9) [30]. The *Salmonella* Pathogenicity Islands (SPIs) present in the genomes were identified using the SPIFinder 1.0 web-based tool (https://cge.cbs.dtu.dk/services/SPIFinder). The virulence genes were annotated by blast searching the amino acid sequences against experimentally verified virulence-associated protein sequences available in the VFDB database [31] with an identity threshold of 80 % and a coverage threshold 80 %.

### Comparative genomics analysis

The Average nucleotide identity calculated by numer algorithm (ANIm) values between the genomes were calculated using pyani.py (https://github.com/widdowquinn/pyani) [32]. The pan genome and core genome were estimated using the OMCL algorithm implemented in the get_homologues package (ver. 3.3.2) [33, 34] (with parameters -S 75 %, -t 0). The orthologous groups generated by get_homologues were used to estimate the pan-genome size using PanGP ver. 1.0.1 [35] with the distance guide (DG) algorithm; the fitted models were also determined. The fitting model for the pan-genome profile curve is described as $y=Ax^{B}+C$ , where *y* is the pan-genome size, *x* is the genome number, and A, B, and C are fitting parameters; the fitting model for core-genome profile curve is described as $y=De^{Ex}+F$ , where *y* is the core genome size, *x* is the genome number, and D, E, and F are fitting parameters. The pan-genome phylogenetic tree was constructed using the get_homologues package (ver. 3.3.2). The presence and absence matrix of the gene families along with the phylogenetic tree, were uploaded to the GLOOME server [36] for gene gain and loss analysis. The high probability of gain and loss events (≥0.8) estimated by the stochastic mapping method were also analysed. The phylogenetic tree was reconstructed using FastTree2 [37] based on the selected core genes by using get_phylomarkers package [33, 34].

### Construction of a gene deletion mutant strain

The *dam* gene, located in a conserved prophage specific to the 16 swine-associated *S*. Derby ST71 strains, was deleted using a double exchange homologous recombination with a suicide vector pDM4, as described previously [38]. A representative strain, S35, was also used in this experiment. The primers used for construction of the gene deletion mutant are listed in Table S2. The generated mutants were verified by PCR and sanger sequencing.

### Motility assays for the WT and *Δdam* strains

The S35 wild-type (WT) strain and *Δdam* mutant were cultured in LB medium overnight, before being transferred to fresh medium for continued culture for an additional 6 h. The cultures were washed twice and diluted in fresh LB medium to ensure an OD_600_ of 1.0. In total, 5 µl of the bacterial cultures was dropped onto the centre of the LB soft‐agar plates (0.3 % agar), and the diameter of the migration zone was measured after 8 h of incubation at 37 °C.

### Adhesion and invasion assays for IPI-2I cells

The porcine IPI-2I intestinal epithelial cells were propagated in DMEM (Basal Media, Shanghai, China) containing 10 % fetal bovine serum. The cells were seeded into 24-well plates at a concentration of 4×10^5^ cells per well, and cultured overnight at 37 °C in 5 % CO_2_. The *S*. Derby S35 WT and *Δdam* strains were grown until stationary phase at 37 °C in LB medium, and subsequently added to each well with a Multiplicity of infection (MOI) of 20 : 1. The cell cultures were incubated at 37 °C in 5 % CO_2_ for 1 h, after which the cultured cells were washed with DPBS (Gibco, Grand Island, NY, USA) three times and solubilized with 1 % Triton X-100 for 5 min; finally, the adhered bacterial cells were counted. For invasion, 100 µg ml^−1^ of gentamycin was added to the cell cultures to lyse the extracellular bacteria after washing, before culturing them for an additional 1 h at 37 °C in 5 % CO_2_. After washing and solubilizing, the solution was serially diluted and the appropriate dilutions were coated onto the LB plates to calculate the number of bacteria. Data were analysed using the two-tailed Student’s *t*-test.

## Results

### The pan-genome of *S*. Derby ST71 strains exhibits an open nature

Previous studies have reported that poultry samples, and not swine samples, were the main sources of the isolated of *S*. Derby ST71 strains based on samples collected in France and Germany [39–41]. Here, multiple *S*. Derby ST71 strains were isolated from a pig slaughterhouse and retailed pork samples in the Jiangsu Province, China. Notably, no poultry-originating ST71 strains were identified in our *S*. Derby strain collection, containing more than 400 *S*. Derby strains collected between 2009 and 2016 [2, 22, 42]. In order to gain insights into the mechanisms underlying the difference in host ranges between the ST71 strains isolated here and previous reported strains, 15 representative *S*. Derby ST71 strains (14 from retailed pork sample and one from a pig slaughterhouse sample) from our *S*. Derby strain collection [22] were sequenced. Comparative genomics analyses were performed with the 65 *S*. Derby ST71 genomes (one swine-associated and 64 poultry-associated) available in the NCBI Refseq database (access on 2 January 2020). The ANIm values of these 80 strains were found to be between 99.86 and 99.99 %, demonstrating highly conserved genomic backgrounds among these strains. Interestingly, we observed that the 16 swine-associated ST71 strains (15 strains sequenced in this study and BCW_2839 (refSeq GCF_002063355.1), a strain isolated from pork samples sold in rural market, Jiyuan, China in 2006) harbour a higher number of genes (4559.56±4, mean±SE, *n*=16) than those in poultry-associated ST71 strains collected in France (4437.38±4.03, *n*=64) (*P*<1e^−5^, two-tailed *t*-test), which motivated us to perform pan-genome analyses, aiming to identify specific genomic regions in the two strain groups and investigate their potential functions.

**Fig. 1.**
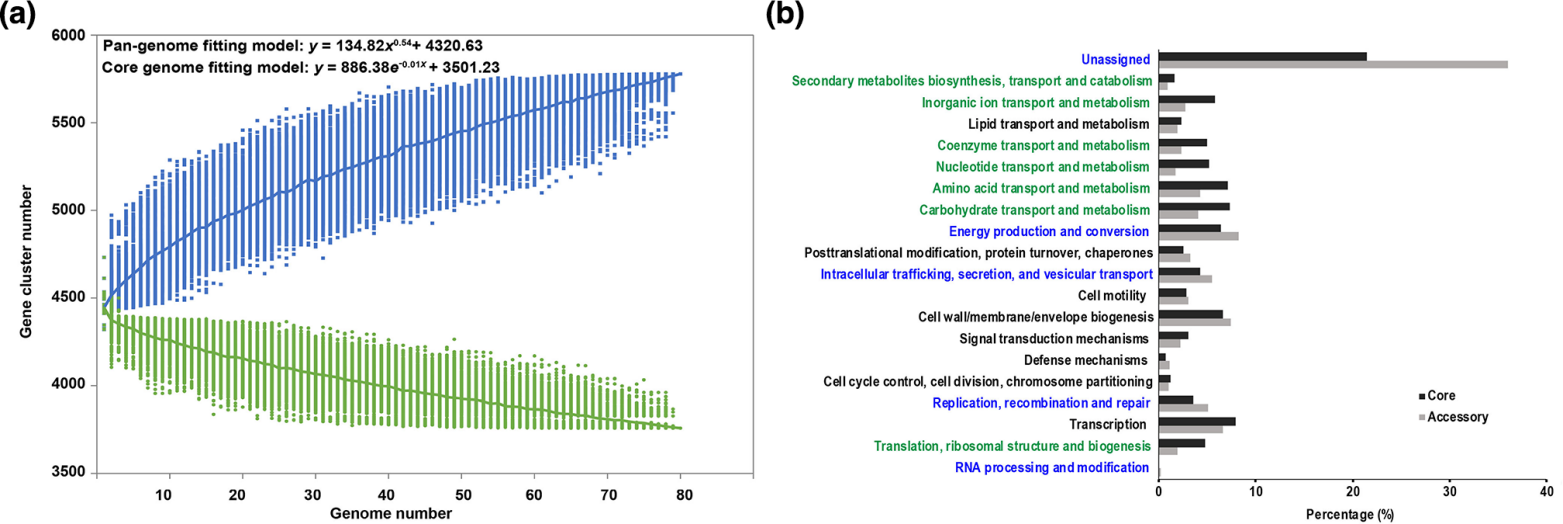
Core and pan-genome analyses of *S*. Derby ST71 strains. (**a**) Estimation of the *S*. Derby pan- and core genome size. Rarefaction analysis demonstrates that the average number of gene clusters in the pan-genome increases while the conserved core gene clusters reaches a plateau as more genomes are analysed. (**b**) Functional distribution comparison between the core and accessory genes of the *S*. Derby strains. The COG categories with significantly different proportion between the two groups were blue coloured (higher in accessory gene group) or green coloured (higher in core gene group) (Fisher exact test, *P*<0.05).

The core genome (i.e. gene families present in all the 80 strains) comprised 3655 orthologous clusters, whereas the pan-genome contained 5778 gene families. The depletion curve and the fitting model for the core genome clearly demonstrated that the number of core *S*. Derby ST71 genes slightly decreased and tended to reach a constant number (parameter E = −0.01; F=3,501.23, 95 % C.I.: 3,501.23±0.15) (model R^2^ >0.996), while the pan-genomes of *S*. Derby ST71 strains were found to be open, as evidenced by the accumulation curve and the fitting parameters (B=0.54, A=134.82±0.1(95 % C.I.)) (model R^2^ >0.999) (Fig. 1a), suggesting that novel gene families will be persistently identified upon inclusion of additional strains into the analysis. Compared to the orthologous clusters in the core genome, the 2123 accessory gene families (i.e. gene families not harboured by all the 80 strains) were underrepresented in the COG categories involved in central metabolism; however, they were overrepresented in COG categories including ‘Intracellular trafficking, secretion, and vesicular transport’, ‘Energy production and conversion’, and ‘Replication, recombination and repair’ (Fig. 1b) (Fisher exact test, *P*<0.05). This indicated the potential importance of these functions in host and/or environmental adaptation of *S*. Derby ST71. For instance, several genes inside the *hyc* gene cluster, involved in Hyc enzyme synthesis and affiliated with ‘Energy production and conversion’, were identified as accessory genes. Although the Hyc enzyme is involved in adaptation to acidic environments, it appears to not be important for pathogenicity in *Salmonella* [43]. Several integrase encoding genes affiliated with ‘Replication, recombination and repair’, such as *xerC* and *xerD*, were identified in the accessory genes, these *xer* genes are known to be involved in horizontal gene transfer (HGT) and play an important role in the ecological niche and host adaptation [44, 45].

### Known *Salmonella* pathogenicity island (SPI) and virulence factor (VF) contents could not differentiate between the poultry- and swine-associated *S*. Derby ST71 groups

**Fig. 2.**
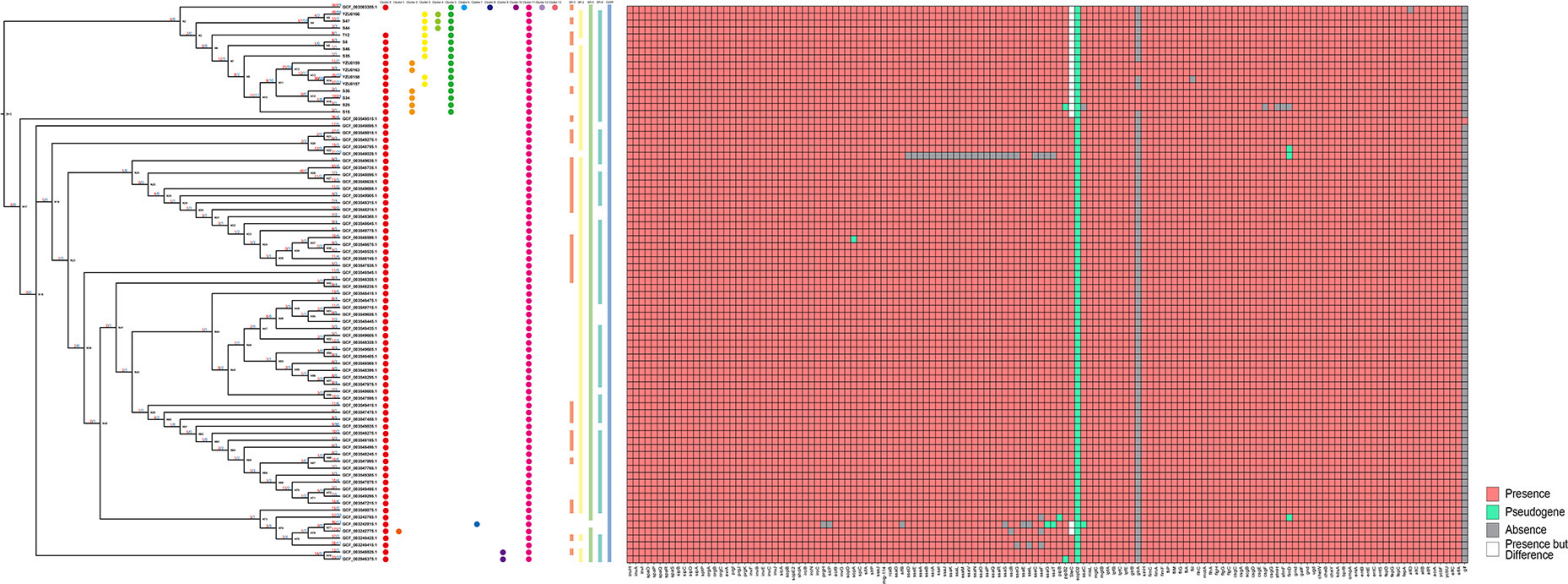
Pangenome and genomic feature analysis of the 80 *S*. Derby ST71 strains. Left panel, phylogenetic tree reconstructed based on the pangenome gene presence/absence data. The 16 swine-associated strains were grouped together and formed a separate cluster from those poultry-associated strains. Red and blue labelled numbers on the branches of the tree denoted number of gene gain and loss events occurred on the branch. Middle panel, the circles and the strips with different colours denote presence of each of prophage clusters and SPIs, respectively. Right panel, the red squares indicated presence of virulence genes, green coloured ones denote pseudogenes, grey denotes gene absence, white denotes gene presence but with gene length difference.

*Salmonella* pathogenicity islands (SPIs) are clusters of genes that are responsible for encoding the various virulence factors (adhesion, invasion, toxin genes, *etc*.) [46]. Five known SPIs, including SPI-1, SPI-2, SPI-3, SPI-4, and C63PI, were identified from the 80 strains using SPIFinder analysis (Fig. 2). C63PI, known to encode protein constituents of the manganese (II) and iron (II) uptake systems [47], was found to be present in all 80 strains, suggesting the importance of C63PI for *S*. Derby ST71. SPI-1, SPI-2, SPI-3, and SPI-4, which are known to encode proteins of the secretion systems and associated effectors in *Salmonella* [48–51] were identified in 37, 73, 79, and 67 strains, respectively, indicating that these SPIs were not critical for *S*. Derby ST71 to occupy the host niches. Notably, the SPI presence/absence patterns could not differentiate between the poultry- and swine-associated *S*. Derby ST71 groups.

In total, 139 VF encoding genes were identified from the 80 *S*. Derby ST71 strains; the distribution patterns of the virulence genes are shown in Fig. 2. Of these VFs, 102 were found to be present in all 80 strains, with eight VFs pseudogeneticized. Pseudogenization of *sopD2*, which is known to contribute to the systemic virulence of *Salmonella* [52] was found in all 80 strains, whereas pseudogenization of the other seven VFs was only identified in a small fraction of strains. While majority of the 80 strains harboured full length of *steC* gene, truncated *steC* was identified in two poultry-associated strains (Fig. S1); however, *steC* has not been reported to be associated with host range determination of *Salmonella* [53, 54]. Thirty-seven VFs were identified in a fraction of the 80 strains, while 35 of them were harboured by majority (>75) of the 80 *S*. Derby ST71 strains, *gtrA* (five strains) and *glf* (one strain) were present in only a small number of strains (Fig. 2, Table S3); however, no consistent VF presence/absence patterns were observed in either group, indicating that the observed host range difference between the two strain groups could not attributed to the known VFs.

### Prophage contents of poultry- and swine-associated *S*. Derby ST71 groups were distinct

The pan-genome phylogenetic tree could clearly separate the swine-associated strains from the poultry-associated strains, forming two distinct clades (Fig. 2). In order to identify clade-specific genes that might contribute to the observed host range differences between the swine-associated and poultry-associated strain groups, gene gain/loss analysis was performed using the GLOOME server, which identified 2110 gene gain events and 1055 gene loss events were identified across the tree. Most of these events were found to occur on the external nodes (i.e. the strains, accounting for 81.1 % of the total gain events and 75.2 % of the total loss events) and not on the internal nodes (both *P* values lower than 0.05, two tailed *t*-test). Importantly, although gene loss events occurred at similar rates for the swine-associated group (on average 13.56 events/strain, *n*=16) and the poultry-associated strains (on average nine events/strain, *n*=64) (*P*=0.24), the swine-associated strains had experienced more gene gain events (on average 39 events/strain) than the poultry-associated strains (on average 17 events/strain) (*P*<0.05, two tailed *t*-test).

**Fig. 3.**
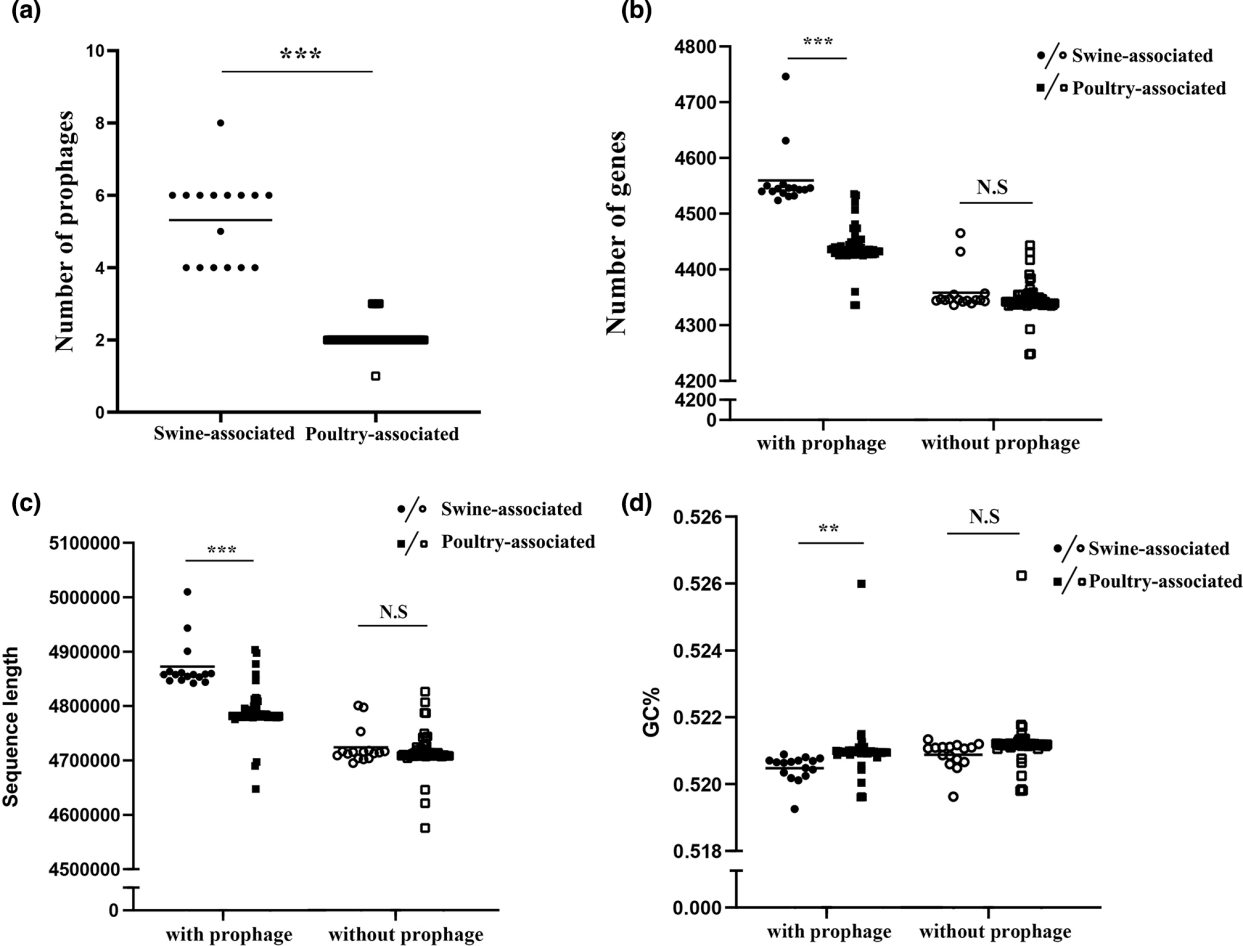
Genomic characteristics comparison between swine-associated and poultry-associated strains. Circles represented swine-associated strains, and squares denoted poultry-associated strains. (**a**) Prophage number comparison between swine-associated and poultry-associated strain groups. (**b**) Gene number comparison between swine-associated and poultry-associated strain groups, with prophage-originated genes included (solid symbols) or removal (hollow symbols). (**c**) Whole genome length comparison between swine-associated and poultry-associated strain groups, with prophage-originated genes included (solid symbols) or removal (hollow symbols). (**d**) GC content comparison between swine-associated and poultry-associated strain groups, with prophage-originated genes included (solid symbols) or removal (hollow symbols).

Interestingly, several prophage-associated genes were identified in the gene-gain families of swine-associated clade, including genes encoding Phage terminase, Phage late control gene D, and Phage integrase, which prompted us to compare the prophage contents between the two strain groups. In total, 217 prophage sequences were identified. Notably, the swine-associated strains were found to harbour significantly more prophages compared with those in the poultry-associated group (5±3 for swine-associated group, *n*=16, and 2±1 for poultry-associated group, *n*=64, respectively) (*t*-test, *P*<0.05) (Fig. 3a). Importantly, the number of remaining genes in the genomes was similar between the two groups (4358±36.10 for swine-associated group and 4346±28.48 for poultry-associated group) once the prophage sequences were removed (*P*>0.1) (Fig. 3b). Similarly, the sequence length and GC content differences observed between the two groups were insignificant after removal of the prophage sequences from the genomes (Fig. 3b, c). Furthermore, the pan-genome phylogenetic tree, constructed based on genomic genes without including prophage-originating genes, as well as the core-genome phylogenetic tree both displayed incongruent topologies compared to the tree constructed based on genomic genes along with prophage-originating genes (Fig. 2, S2, and S3), where the swine-associated and poultry-associated strains exhibited a mixed structure (Fig. S2). Therefore, we concluded that the prophage is the main driver that mediates genome size and content differentiation in the *S*. Derby ST71 population.

The 217 prophage sequences identified from the 80 strains were clustered to 14 prophage sequence groups, with prophage cluster11 observed in all strains and cluster1 identified in majority of the 80 strains (Fig. 2), which indicated that these two prophages were acquired at a relatively ancient timepoint by the *S*. Derby ST71 population. The remaining 12 prophage sequence groups were present only in relatively smaller fractions of strains, with most specifically present in swine-associated strains, but rarely identified in poultry-associated strains. Notably, the insertion sites of all the prophage groups identified in swine-associated strains were found to be strain-specific and highly diverse, indicating that the prophage acquisition events occur independently for these strains after their differentiation from the last common ancestor.

### Functional analysis of the prophage-originating genes involved in niche adaptation

**Fig. 4.**
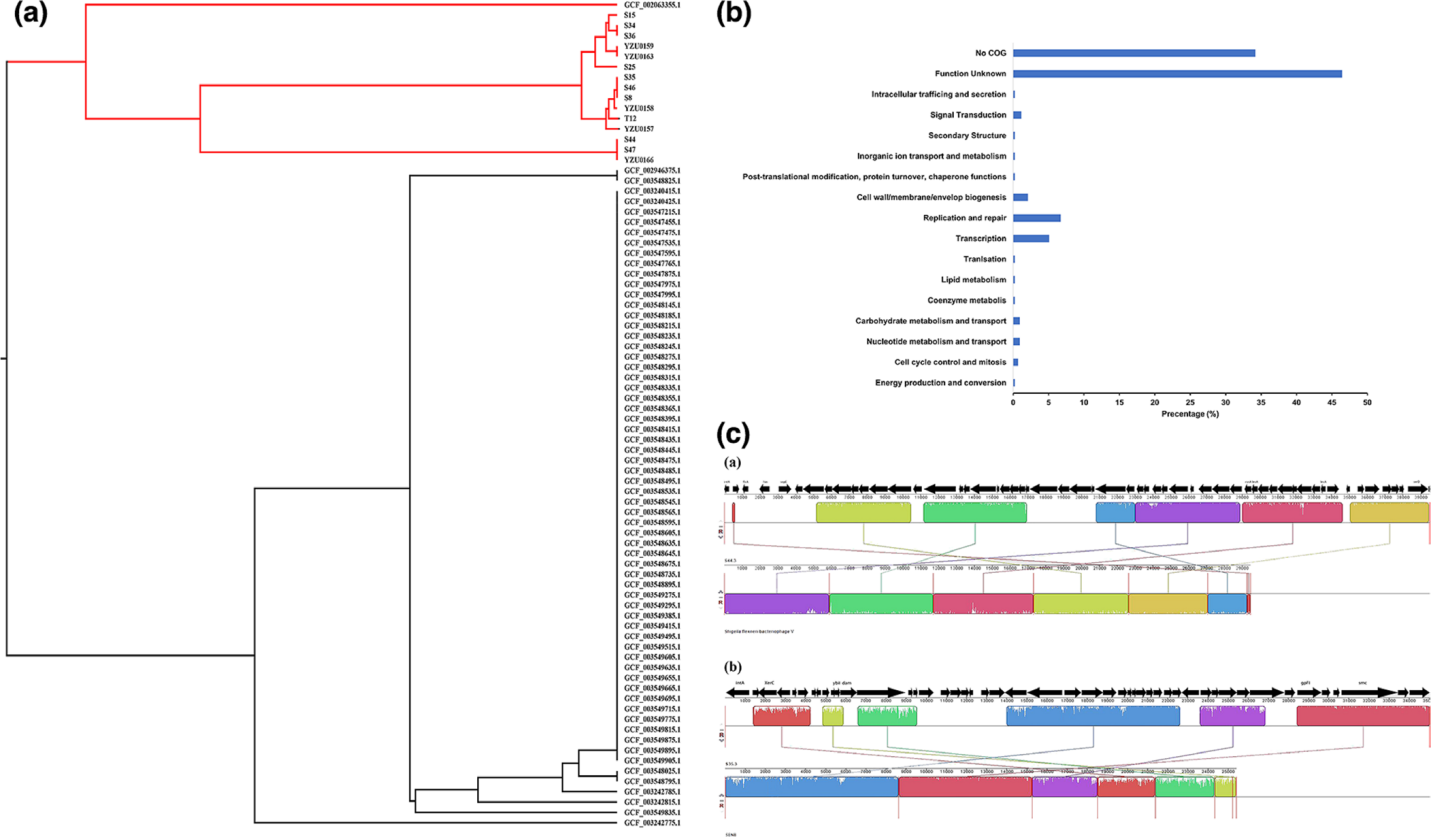
(**a**) The phylogenetic tree reconstructed based on gene presence/absence of the prophage gene families (**b**) Functional distribution of the prophage gene families. (**c**) Sequence alignments between the representative prophage sequences (up) and their relatives (top hit from NCBI database) (down). The sequences are aligned using Mauve, and local collinear blocks within each alignment are represented as blocks of similar colour connected with lines.

Between 67 and 281 genes were predicted from the prophage sequences in each strain, with swine-associated strains harbouring significantly higher number of prophage-originating genes (201.3±5.44 genes per strain) than those in poultry-associated strains (91.4±1.25 genes per strain) (*P*<1.45e-44, two-tailed *t*-test). The prophage-originating genes were grouped into 394 gene families, and the phylogenetic tree constructed based on these gene families could clearly distinguish the swine-associated strains from the poultry-associated strains (Fig. 4), which was consistent with the whole-genome based phylogenetic tree (Fig. 2). Only a small fraction of gene families were COG annotated and classified into ‘Replication and repair’, ‘Transcription’ as well as other several COG categories (Fig. 4). Most of these gene families could not be assigned clear functional annotations, suggesting that the cargo genes harboured in the prophage sequences could serve as a functional resource to discover new genes.

Prophages can carry functional genes that benefit the bacterial host, including virulence factor encoding genes [55]. The *yfdH* gene was identified in all 80 strains, which is a cargo gene from cluster11. The product of the *YfdH* gene is involved in modification of lipid A, which is essential for resistance to polymyxin and cationic antimicrobial peptides. We also identified a *sopE*-containing prophage cluster, cluster4, distributed in a clade containing three swine-associated strains. The representative sequence of prophage cluster4, S44.3, revealed the best blast hit to *Shigella flexner* bacteriophage V (NCBI accession no. U82619.2) with a 98.32 % similarity, suggesting that phages can infect different bacterial species and result in broad bacterial host ranges [56]. Although the backbones of S44.3 and *Shigella flexner* bacteriophage V are highly conserved, several S44.3-specific genes were identified, including the *sopE* gene (Fig. 4). The *sopE* gene was not identified in any other strain, except in GCF_002063355.1, which is also a swine-associated strain. *SopE* is a known guanidyl exchange factor (GEF) that can be translocated into infected animal and human cells by the SPI-1 encoded type III secretion system, thereby promoting efficient entry of the pathogen into host cells [57–59]. Interestingly, prophage cluster5 was found to be present in all 16 swine-associated strains; however, it was absent in the poultry-associated clade. The diverse insertion sites of this prophage among the genomes also suggested that it is independently acquired by the 16 strains recently. The representative prophage sequence in cluster5, S35.3, exhibited 94.57 % sequence similarity to a known prophage, *Salmonella enterica* subsp. *Salamae* SEN8 (NCBI accession no. NC_047753.1); however, it harbours several extra regions. Inside the prophage S35.3-specific region, several genes, including the *dam* gene (encoding a DNA adenine methylase) were identified (Fig. 4). A previous study has revealed that the *dam* gene is crucially involved in *Salmonella* host infection and virulence regulation [60]. However, the *dam* gene has been previously reported to be chromosome-inherent for *Salmonella *, which was also identified in all 80 investigated strains (identical to STM3484 gene, *dam*). Dam encoding genes located in the prophage cluster5 sequences were observed to form a separate clade among the 80 *dam* sequences (Fig. S4).

### The *dam* gene encoded by a prophage specific to and conserved in swine-associated *S*. Derby ST71 strains contributes to cell adhesion ability of the *S*. Derby ST71 strains

**Fig. 5.**
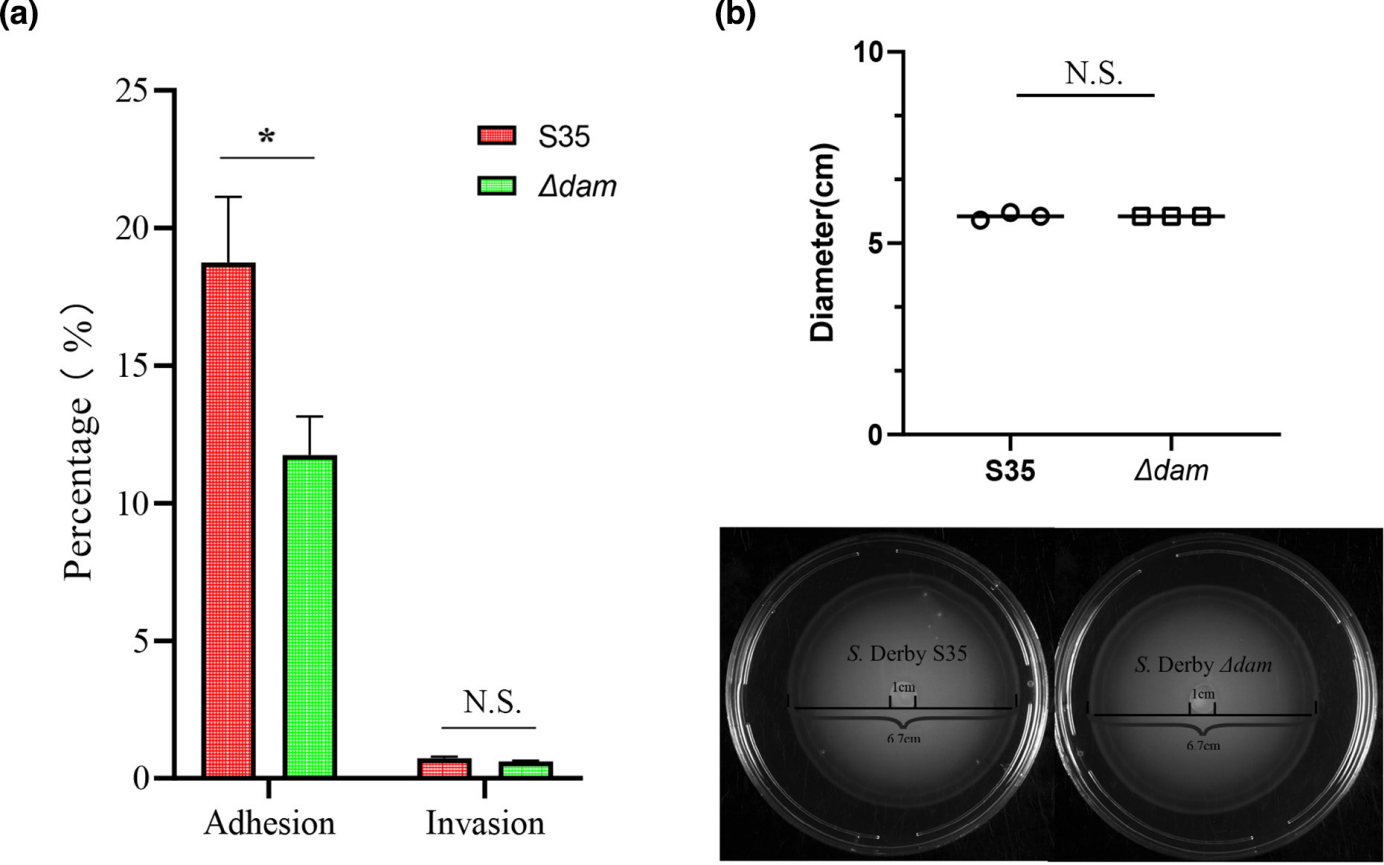
(**a**) Relative intracellular loads at 1 h (adhesion) and 2 h (invasion) post-infection of *S*. Derby S35 and its mutants *Δ*dam in IPI-2I. The formula for calculating the adhesion/invasion rate of bacteria to cells: (the number of adherent/invading bacteria to cells / initial number of bacteria in the hole)×100 %. (**b**) Motility assays of WT and deletion mutant strain. Diameter of mobility formula: large circle diameter - small circle diameter. The difference was analysed by *P*-values from two-tailed Student’s *t*-test (*P<0.05, *P*>0.05 not significant).

The biological function of the prophage carrying the *dam* gene specifically identified in the swine-associated strains was determined by constructing a gene deletion mutant and performing bacterial phenotype and *in vitro* infection assays. The results demonstrated that the *dam* gene does not affect the growth characteristics and motility of the *S*. Derby S35 strain (Fig. 5). However, the prophage carrying the *dam* gene endows the host strain with 59.57 % higher adhesion ability onto the host epithelial cells compared to other strains (*P*<0.05) (Fig. 5). These results further suggested that prophages likely function as vehicles to carry functional genes that endow the host bacteria advantages to adapt challenging environments and expand its niches [61].

## Discussion

In this study, we sequenced the genomes of 15 representative swine-associated *S*. Derby ST71 strains selected from our *S*. Derby strain stocks collected between 2009 and 2016 [2, 22, 42], and performed gene content-based comparative genomics analysis with the publicly available *S*. Derby ST71 strains that were mainly poultry-associated. The size of core genome of *S*. Derby ST71 strains (3655 gene families) was larger than the estimated conserved core genome size (~3200 genes) of *Salmonella enterica* species [62], which indicated the presence of a lineage-specific core genome in *Salmonella* [63]. Notably, the pan-genome of *S*. Derby ST71 population was found to exhibit an open nature (Fig. 1). The pan-genome is shaped by drift and selection of constant gene gains and losses in the genome, and the selection pressures are mainly derived from the niche bacteria colonize [64]. Thus, some accessory genes may only provide the bacterial host benefits and maintained in specific niches. Previous studies have demonstrated that *S. enterica* subsp. *enterica* harbours an open pan-genome with large number of accessory genes identified, and the accessory genes are probably beneficial for this pathogen given the highly variable host range ranges and environments members in this taxon live in [10, 11]; the pan-genome of strains affiliated with the same serotype of *S. enterica* subsp. *enterica *, whose genome are more conserved compared with the entire *S. enterica* subsp. *enterica *, is found to be open for certain serotypes that are widely-distributed in many environments and have a wide host range or closed for certain host-specialized serotypes such as *S*. Typhi [10, 11, 65]. Our results demonstrated that the pan-genome of strains derived from even a single ST lineage in the same *Salmonella* serotype but with distinct host range was open, further indicating that the rich genetic contents in the accessory genome play an important role in niche and host adaptation for the pathogens [66–68]. Indeed, several genes involved in niche adaptation, such as the Hyc enzyme synthesis-associated genes, were observed in the accessory genome of the *S*. Derby ST71 strains in this study. Moreover, based on the current eggNOG orthology database [27], a large fraction of genes in the accessory genome could not be COG annotated (Fig. 1b). These unannotated genes can serve as a pool to discover novel functions.

Of note, in the functional category ‘Replication, recombination and repair’ that was overrepresented in the accessory gene families, multiple prophage-associated genes, such as Phage terminase, Bacteriophage replication gene A protein (GPA), and Phage integrase, were identified (Table S4), indicating prophages were an important source of the gene families affiliated with ‘Replication, recombination and repair’ in the accessory genome. We identified 217 prophages from the 80 *S*. Derby ST71 strains, and further observed that the swine-associated strains harboured significantly more prophages compared with the poultry-associated strains (Fig. 3). Importantly, these prophages were found to be the main driver causing the observed gene number and genome size differences, as well as the distinct accessory gene distribution patterns between the swine- and poultry-associated groups (Figs 2 and 3). Prophages, which are derived from the phages that infect the bacterial host, have been reported as one of the main components of the accessory genome of many bacterial species, and are recognized as essential drivers of bacterial genomes’ evolution and differentiation [20, 69, 70]. Previous studies have demonstrated that significantly higher populations of phages are present in environment samples from pig farms compared with those from poultry farms [58, 59], indicating the occurrence of environment-associated bacteria-phage coevolution for the two ST71 populations. The acquired prophages can either be maintained in the bacterial chromosome or drift away, and those containing beneficial cargo genes that contribute to niche adaptation of the bacterial host are more likely persistently present in the evolution [20, 69, 71]. Indeed, a relatively ancient and stably maintained prophage by all the 80* S*. Derby ST71 strains, cluster11, was found to harbour a cargo gene *yfdH*, which plays an important role in resisting against antimicrobial compounds such as polymyxin and cationic antimicrobial peptides for the bacterial host (Fig. 2). Importantly, we found that a prophage sequence that was specifically present in the 16 swine-associated strains contained a *dam* gene, and the gene deletion-based experiments demonstrated that this prophage-carried *dam* gene was beneficial for *S*. Derby to invade the swine host by increasing the cell adhesion ability onto the porcine intestinal epithelial cells (Figs 2, 4 and 5). Furthermore, a prophage-carried type III effector, *sopE*, which can promote efficient entry of the pathogen into host cells, was identified in several swine-associated strains but not in the poultry-associated strains (Figs 2 and 4). These results indicated that prophages contributed to niche and host adaptation of the *S*. Derby ST71 strains. Given that majority of genes in the prophages are still not obtained functional annotations based on current database yet (Fig. 4), the roles of prophages in the genome differentiation and niche adaptation need to be further explored.

Overall, 15 representative swine-associated *S*. Derby ST71 strains isolated from China we sequenced, which revealed the genomic features associated with the differentiation between swine-originating strains and previous reported poultry-specific *S*. Derby ST71 strains through comparative genomics analyses. We found that prophages are an important source for the genome expansion of swine-originating *S*. Derby ST71 strains, resulting in significantly larger genome sizes, higher number of genes, and lower GC contents in the swine-originating *S*. Derby ST71 strains compared with those in reported poultry-specific strains. Several cargo genes inside the prophages that were specifically harboured by the swine-associated strains, including *sopE* and *dam*, are likely beneficial to the bacterial hosts for niche adaptation. Our gene deletion experiment further demonstrated that the *dam* gene is important for adhesion of *S*. Derby onto host cells. This study provides novel insights into the roles of prophages during the genome differentiation of *Salmonella* populations.

## Supplementary Data

Supplementary material 1

Supplementary material 2
