# Supplementary material for: Prophage-mediated genome differentiation of the Salmonella Derby ST71 population
Source: Microb Genom. 2022 Apr 22;8(4):000817. doi: 10.1099/mgen.0.000817 (PMC9453062; doi:10.1099/mgen.0.000817)
Supplement: Supplementary material 1 [file mgen-8-0817-s001.pdf]

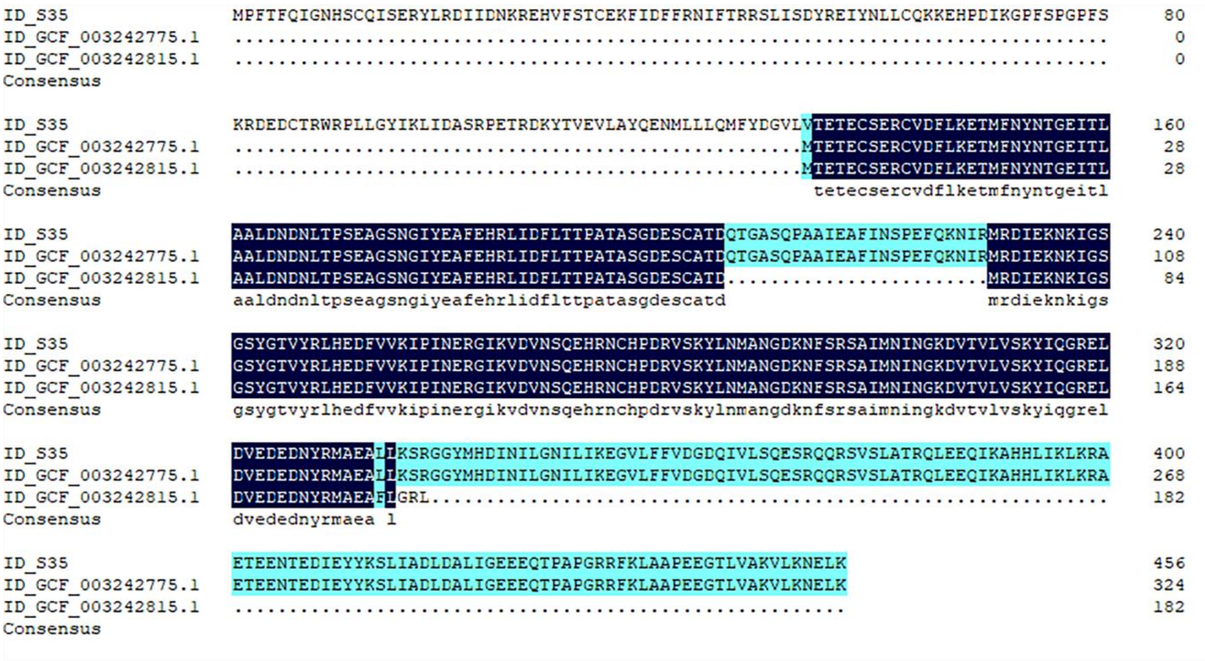

Supplementary Figure S1. The difference in the sequence length of SteC in two poultry-associated strains. ID_S35 denotes the full length of SteC (compared with the sequence from *Salmonella enterica s*ubsp. *enterica* serovar Typhimurium str. LT2 (VFDB accession number VFG042066(gb|NP_460656)), and the remaining two sequences are the truncated *steC* sequences harbored by two poultry-associated strains.

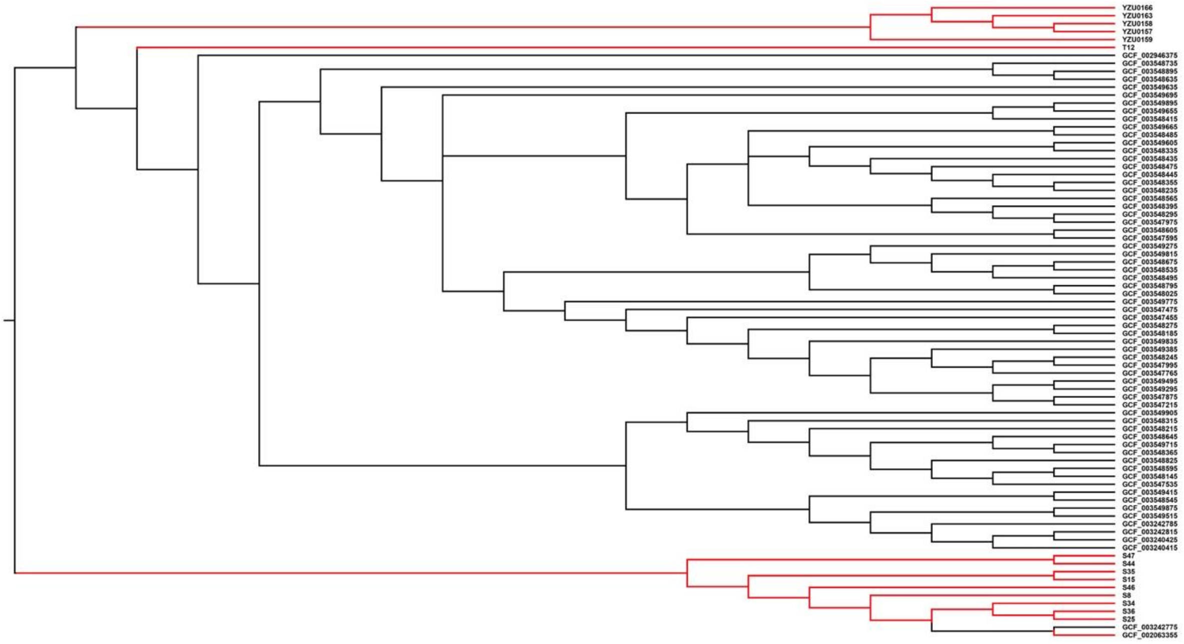

Supplementary Figure S2. Phyletic tree reconstructed based on pan-genome gene presence/absence data with prophage-originating genes removed. The swine-associated strains were red labeled.

Company Limited by Guarantee l Registered in England No. 1039582 l Registered Office Charles Darwin House, 12 Roger Street, WC1N 2JU, UK
Registered as a Charity: 264017 (England & Wales); SC039250 (Scotland)
633bf4b3-e353-4351-8377-7453bfb0447c

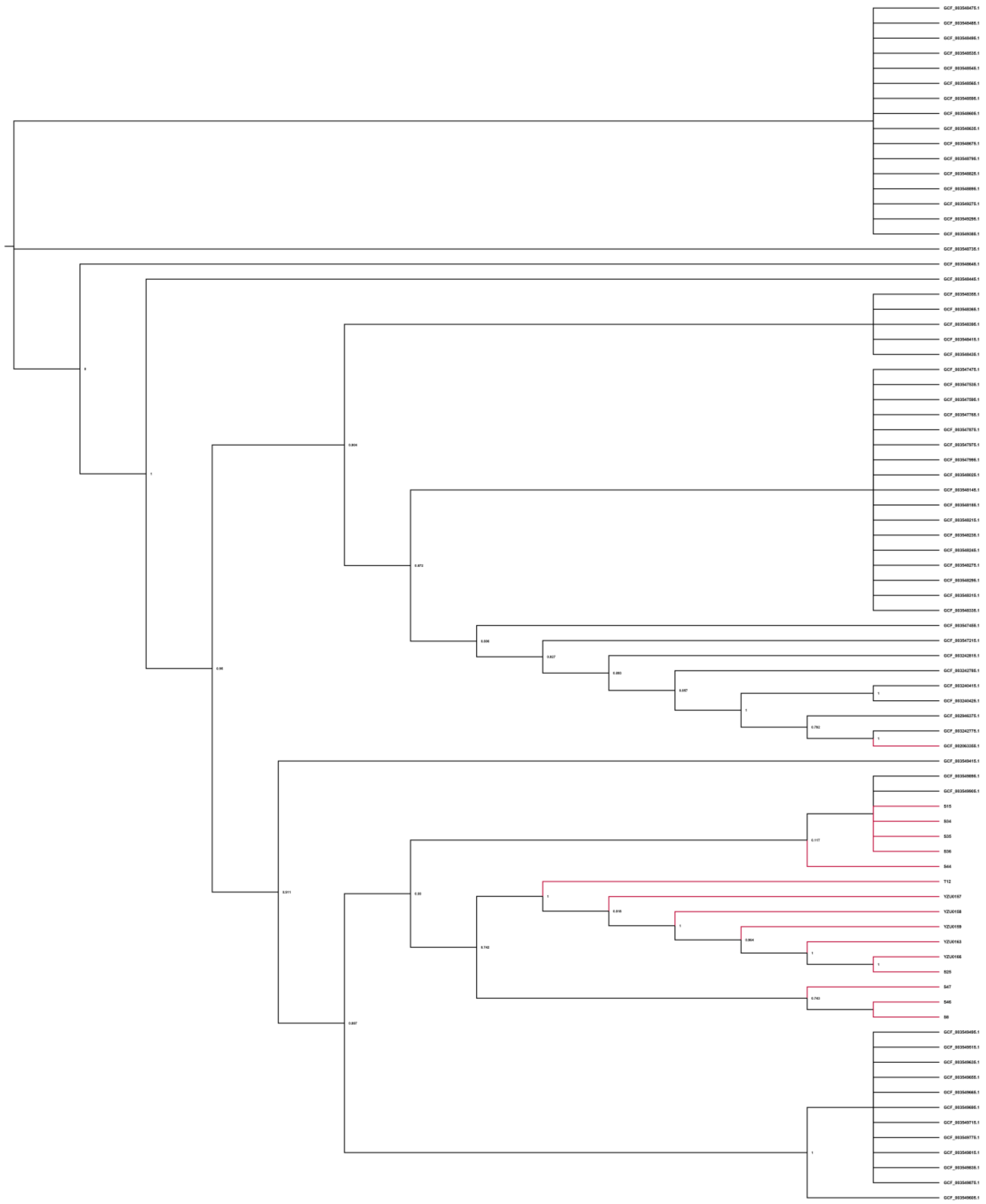

Supplementary Figure S3. Phylogenetic tree reconstructed based on the concatenated sequences of 196 core genes selected by Get_phylomakers package. The swine-associated strains were red labeled.

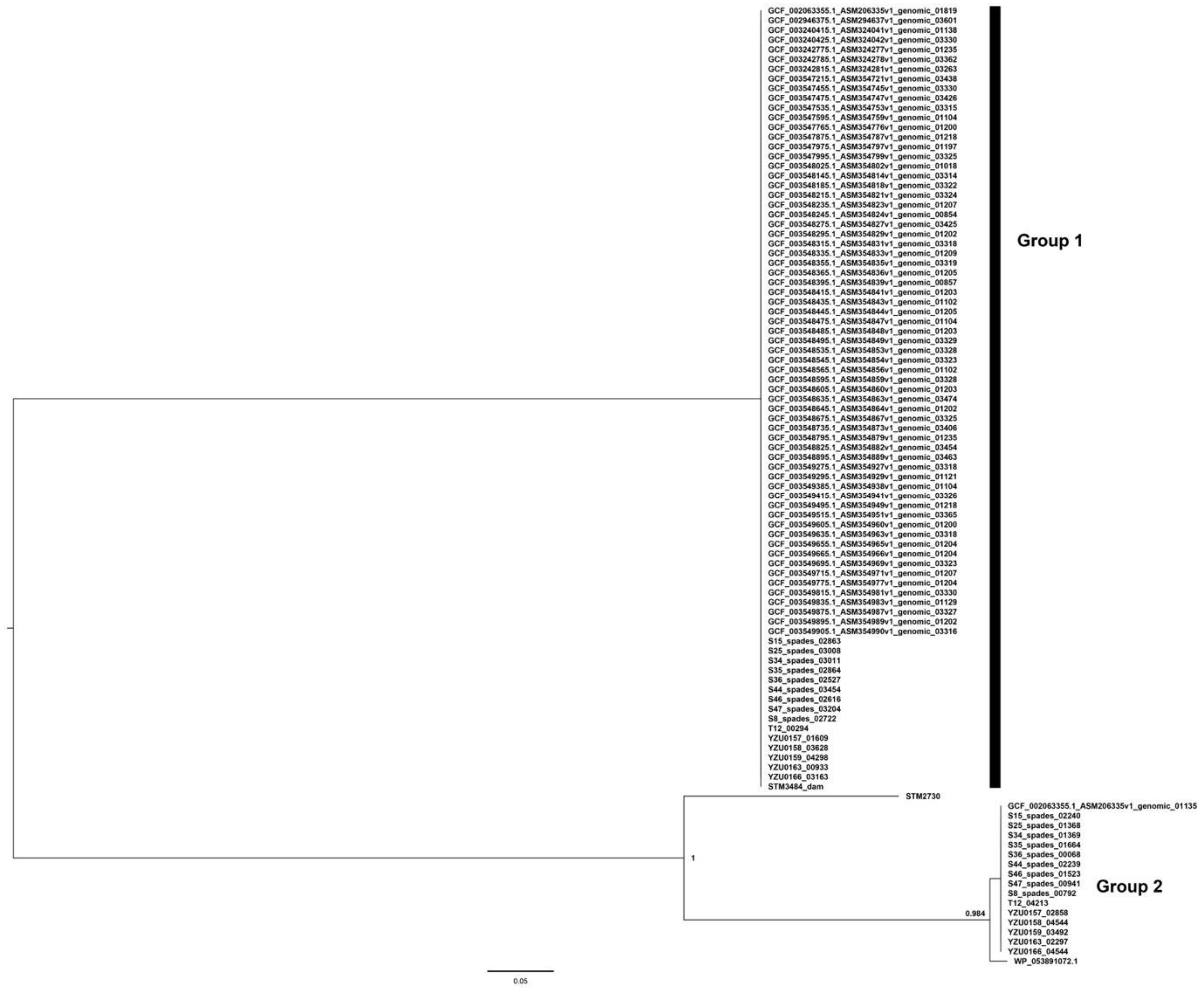

Supplementary Figure S4. Phylogenetic tree of dam gene families (constructed using protein sequences, fasttree, wag gamma).

Table S1. *Salmonella* Derby strains used for comparative genomics analysis in this study.

ID	geographic location	isolation source	collection date	reference
S15	China	Swine	2014	This study
S25	China	Swine	2014	This study
S34	China	Swine	2014	This study
S35	China	Swine	2014	This study
S36	China	Swine	2014	This study
S44	China	Swine	2013	This study
S46	China	Swine	2014	This study
S47	China	Swine	2015	This study
S8	China	Swine	2014	This study
YZU0157	China	Pig slaughterhouse	2014	This study
YZU0158	China	Swine	2014	This study
YZU0159	China	Swine	2014	This study
YZU0163	China	Swine	2014	This study
YZU0166	China	Swine	2015	This study
T12	China	Swine	2015	This study
GCF_002063355.1	China	Swine	2006	ncbi
GCF_002946375.1	France	Poultry	2014	[1]
GCF_003240415.1	France	Human	2015	[2]
GCF_003240425.1	France	Human	2015	not available
GCF_003242775.1	France	Human	2014	[2]
GCF_003242785.1	France	Human	2015	[2]
GCF_003242815.1	France	Human	2014	[2]
GCF_003547215.1	France	Poultry	2015	[2]
GCF_003547455.1	France	Poultry	2015	[1]
GCF_003547475.1	France	Swine	2015	[1]
GCF_003547535.1	France	Poultry	2015	[1]
GCF_003547595.1	France	Poultry	2015	[1]
GCF_003547765.1	France	Poultry	2015	[1]
GCF_003547875.1	France	Poultry	2014	[1]
GCF_003547975.1	France	Poultry	2014	[1]
GCF_003547995.1	France	Swine	2014	[1]

GCF_003548025.1	France	Swine	2014	1
GCF_003548145.1	France	Poultry	2014	1
GCF_003548185.1	France	Poultry	2014	1
GCF_003548215.1	France	Poultry	2014	1
GCF_003548235.1	France	Poultry	2014	1
GCF_003548245.1	France	Poultry	2014	1
GCF_003548275.1	France	Poultry	2014	1
GCF_003548295.1	France	Poultry	2014	1
GCF_003548315.1	France	Poultry	2014	1
GCF_003548335.1	France	Poultry	2014	1
GCF_003548355.1	France	Poultry	2014	1
GCF_003548365.1	France	Poultry	2014	1
GCF_003548395.1	France	Poultry	2014	1
GCF_003548415.1	France	Poultry	2014	1
GCF_003548435.1	France	Poultry	2014	1
GCF_003548445.1	France	Poultry	2014	1
GCF_003548475.1	France	Poultry	2014	1
GCF_003548485.1	France	Poultry	2014	1
GCF_003548495.1	France	Poultry	2014	1
GCF_003548535.1	France	Poultry	2014	1
GCF_003548545.1	France	Poultry	2014	1
GCF_003548565.1	France	Poultry	2014	1
GCF_003548595.1	France	Poultry	2014	1
GCF_003548605.1	France	Poultry	2014	1
GCF_003548635.1	France	Poultry	2014	1
GCF_003548645.1	France	Poultry	2014	1
GCF_003548675.1	France	Poultry	2014	1
GCF_003548735.1	France	Poultry	2014	1
GCF_003548795.1	France	Poultry	2014	1
GCF_003548825.1	France	Poultry	2014	1
GCF_003548895.1	France	Poultry	2014	1
GCF_003549275.1	France	Swine	2015	1
GCF_003549295.1	France	Poultry	2015	1
GCF_003549385.1	France	Poultry	2015	1
GCF_003549415.1	France	Poultry	2015	1

GCF_003549495.1	France	Swine	2014	[1]
GCF_003549515.1	France	Poultry	2014	[1]
GCF_003549605.1	France	Poultry	2014	[1]
GCF_003549635.1	France	Poultry	2014	[1]
GCF_003549655.1	France	Poultry	2014	[1]
GCF_003549665.1	France	Poultry	2014	[1]
GCF_003549695.1	France	Poultry	2014	[1]
GCF_003549715.1	France	Poultry	2014	[1]
GCF_003549775.1	France	Poultry	2014	[1]
GCF_003549815.1	France	Poultry	2014	[1]
GCF_003549835.1	France	Poultry	2014	[1]
GCF_003549875.1	France	Poultry	2014	[1]
GCF_003549895.1	France	Poultry	2014	[1]
GCF_003549905.1	France	Poultry	2014	[1]

Table S2. Primers used in this study.

Primer Name	Primer Sequence (5′ to 3′)
dam-up-F	GAGCGGATAACAATTTGTGGAATCCCGGGACATTACCTGTCAGGAAATCGCAGAG
dam-up-R	TTGCGATACATCAGCAGCAACCTTTTTATAGAGGT
dam-down-F	TTGCTGCTGATGTATCGCAATTTCACTCACCACTA
dam-down-R	AGCGGAGTGTATATCAAGCTTATCGATACCCAGCGTACAGAGTGATCAGGTTTGA
dam-out-F	CGGGTATCCCTTTATCTAAATTCAG
dam-out-R	TTAACATCATCGGTGGCGTACAAAT
dam-in-F	CAGGGAGGTGGCTTATTACAACA
dam-in-R	CGCAATACACCACATCTCCCACC

References

1. **Sevellec Y, Vignaud ML and Granier SA, et al.** Polyphyletic Nature of *Salmonella enterica* Serotype Derby and Lineage-Specific Host-Association Revealed by Genome-Wide Analysis. *Front Microbiol* 2018; 9: 891 doi: 10.3389/fmicb.2018.00891.
2. **Sevellec Y, Felten A and Radomski N, et al.** Genetic Diversity of *Salmonella* Derby from the Poultry Sector in Europe. *Pathogens* 2019; 8 doi: 10.3390/pathogens8020046.
